# Mathematical Models of Plasmid Population Dynamics

**DOI:** 10.3389/fmicb.2021.606396

**Published:** 2021-11-04

**Authors:** José Carlos Ramón Hernández-Beltrán, Alvaro San Millán, Ayari Fuentes-Hernández, Rafael Peña-Miller

**Affiliations:** ^1^Center for Genomic Sciences, Universidad Nacional Autónoma de México, Cuernavaca, Mexico; ^2^National Centre for Biotechnology (CNB-CSIC), Madrid, Spain

**Keywords:** mathematical modeling, horizontal gene transfer, microbial ecology, bacterial evolution, plasmid dynamics

## Abstract

With plasmid-mediated antibiotic resistance thriving and threatening to become a serious public health problem, it is paramount to increase our understanding of the forces that enable the spread and maintenance of drug resistance genes encoded in mobile genetic elements. The relevance of plasmids as vehicles for the dissemination of antibiotic resistance genes, in addition to the extensive use of plasmid-derived vectors for biotechnological and industrial purposes, has promoted the in-depth study of the molecular mechanisms controlling multiple aspects of a plasmids’ life cycle. This body of experimental work has been paralleled by the development of a wealth of mathematical models aimed at understanding the interplay between transmission, replication, and segregation, as well as their consequences in the ecological and evolutionary dynamics of plasmid-bearing bacterial populations. In this review, we discuss theoretical models of plasmid dynamics that span from the molecular mechanisms of plasmid partition and copy-number control occurring at a cellular level, to their consequences in the population dynamics of complex microbial communities. We conclude by discussing future directions for this exciting research topic.

## Introduction

Plasmids are self-replicating, extra-chromosomal DNA molecules widely distributed across bacteria. In addition to regulating their replication and copy number in the host cell, thus propagating vertically within the bacterial population, some plasmids can spread horizontally between neighboring bacterial cells and transfer accessory genes that help bacteria to adapt to new environments and stressful conditions (Norman et al., 2009). There are different mechanisms available for the horizontal transfer of plasmids, but conjugation–plasmid transfer between a donor and a recipient cell through a conjugative pilus–is arguably the best-studied (Smillie et al., 2010). The repertoire of plasmid-encoded adaptive genes is extremely diverse, ranging from genes allowing bacteria to decontaminate heavy-metal polluted environments to others promoting the establishment of symbiotic relationships between bacteria and plants (Hall et al., 2015; Wang et al., 2018). From a human perspective, plasmids represent a concern for public health, because they can encode important traits for pathogenic bacteria, such as virulence factors or antibiotic resistance determinants (Partridge et al., 2018; Pilla and Tang, 2018). In fact, it is believed that plasmids are the main drivers of the spread of antibiotic resistance genes among clinically relevant bacteria and have played an important part in the current global antimicrobial resistance crisis (San Millan, 2018).

**FIGURE 1:**
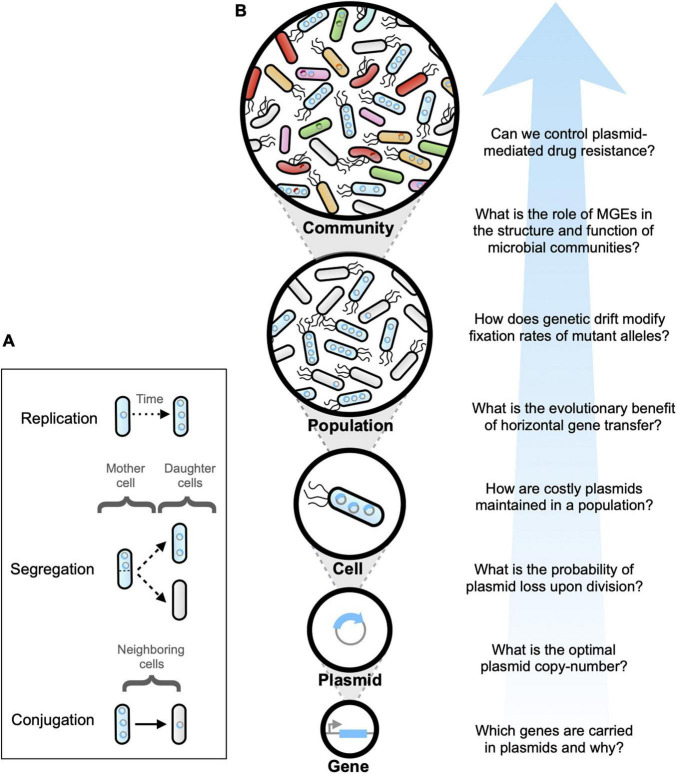
**(A)** Plasmid dynamics emerges as a result of the complex interaction between plasmid replication and copy-number control (occurring inside individual cells), plasmid segregation (from mother to daughter cells), and plasmid conjugation (between neighboring cells). **(B)** Schematic diagram illustrating multiple complexity levels involved in plasmid life cycle. Each complexity level yields important questions that have been addressed using mathematical modeling and computer simulations.

Three main processes determine a plasmid’s lifestyle: (1) replicating until reaching a critical copy-number inside the host cell, (2) partitioning between daughter cells upon division, and, in some cases, (3) transmitting by means of cell-cell contact (see Figure 1A). Therefore, the number of plasmids carried by each cell can be viewed as a time-dependent variable that changes as a function of the molecular interaction between bacterial hosts, plasmids, and the extracellular environment. Hence, it has been argued that replication, partition and transmission of plasmids underlie a complex system that can be studied theoretically using tools developed in the context of dynamical systems and numerical computing.

For instance, a series of theoretical models proposed in the 1980s evaluated the probability of plasmid loss based on the replication mechanism and the mean plasmid copy-number (Nordström et al., 1980; Bremer and Lin-Chao, 1986; Ataai and Shulert, 1987; Keasling and Palsson, 1989). Mathematical modeling also provided an abstract framework to study the regulatory efficiency (Paulsson and Ehrenberg, 2001) and robustness (Brendel and Perelson, 1993) of different plasmid copy-number control mechanisms to environmental perturbations and population heterogeneities. Other early models evaluated the role of noise in the dynamics of plasmid replication (Nordström et al., 1984; Müller et al., 1991; Goss and Peccoud, 1998), the consequences of incompatibility in the stability of plasmids (Ishii et al., 1978; Novick and Hoppensteadt, 1978; Condit and Levin, 1990), and the formation of multimers through homologous recombination, a process known as the “*dimer catastrophe*” (Summers, 1991; Summers et al., 1993). Altogether, these studies greatly improved our understanding of how different molecular copy-number control and partitioning mechanisms determine the probability of segregational loss at the moment of division.

These studies were motivated by developments in molecular biology and bioengineering that used plasmids as vectors for genetic engineering. A problem of using plasmid-derived vectors, however, is that most are unstable in the absence of selection for the traits they encode. That is, there exists a probability higher than zero that a cell inherits no plasmids from its mother. Moreover, if plasmid-free cells have competitive advantage over plasmid-bearing cells, then the subpopulation of cells carrying plasmids will reduce in frequency until driven to extinction. In contrast, if the plasmid encodes for an adaptive gene, then plasmids can be stably maintained in the population through positive selection. Crucially, even when the plasmid carries a beneficial gene, it could be optimal for the host to incorporate this gene into the chromosome, thus rendering the plasmid redundant and susceptible to being lost through purifying selection. This apparent paradox represents an existential problem for plasmids and a fundamental problem for plasmid biology.

From the onset, mathematical modeling played a critical role in addressing the “*plasmid paradox*” (Harrison and Brockhurst, 2012), by defining the impacts of horizontal transmission, spatial structure, fitness cost compensation, gene carriage and interactions within and between hosts, on plasmid persistence in populations and communities. However, despite continuous progress that has spanned over four decades, modeling plasmid dynamics still represents a difficult and ongoing problem. Of course, this is not unexpected, as plasmid replication, transmission and partition are stochastic processes with consequences that span multiple temporal and spatial scales, and therefore are very difficult to study both analytically and computationally. Figure 1B illustrates the multiple levels of complexity involved in biological aspects driving plasmid dynamics: from genes encoded in plasmids to plasmids replicating inside individual cells, that in turn interact with other cells within bacterial populations by transferring plasmids between members of complex microbial communities.

In this review, we explore the myriad of mathematical models available in the field, with an emphasis on discussing different methodological approaches used to study plasmid dynamics in bacterial populations. We also present different approaches used to model horizontal transmission of plasmids between bacterial cells. Briefly, transmission of plasmids in bacterial populations is generally modeled assuming mass action kinetics, namely that the probability of plasmid transfer occurs randomly at a frequency that is jointly proportional to the densities of donor and recipient cells. This assumption allows the use of ordinary differential equations (ODEs) to model plasmid transfer between different sub-populations in well-mixed environments, or partial differential equations (PDEs) to study plasmid transmission both in space and time. We will also discuss other mathematical and computational frameworks used to relax this assumption and study conjugative dynamics in spatially explicit environments, notably discrete-space continuous-time stochastic models and other types of individual-based models (IBMs).

Finally, plasmid biology has historically been a field in which experiments and theory are often combined in the same study (more than half of the modeling studies discussed in this manuscript calibrate parameters or contrast their theoretical predictions with data acquired using experimental model systems, as illustrated in Supplementary Table 1). We will discuss a series of studies that have combined theory with experimental microbiology to study different aspects of plasmid biology; plasmid host-range, plasmid-mediated socio-biological interactions, and the co-evolutionary dynamics of plasmid-host associations, to mention a few. We conclude by describing recent theoretical and computational approaches used to study horizontal gene transfer (HGT) in polymicrobial communities in complex environments, like the soil or the gut microbiota, as well as other open problems in microbial population dynamics.

## Modeling Plasmid Dynamics Inside Bacterial Hosts

Mathematical models have been used to study different aspects of plasmid biology at a cellular level. Of note, the interaction between DNA replication and cell cycle (Kuo and Keasling, 1996), the interaction between plasmid copy-number and the strength of promoters (Lee and Bailey, 1984), and the influence of temperature in plasmid copy-number control (Leipold et al., 1994). But not only have mathematical models been useful in understanding the mechanisms underlying different processes involved in a plasmid’s life cycle (replication, partition, and transmission), they have also probed the validity of different statistical models and identified biologically realistic parameter values. Furthermore, these studies provided evidence that transitions between plasmid-bearing and plasmid-free cellular states are driven by random processes that can be modeled implicitly at a population-level as transition rates between subpopulations, thus establishing the foundations for population dynamic models.

To model the interaction between plasmid segregation and replication, several studies (Nordström et al., 1984; Seo and Bailey, 1985; Boe and Rasmussen, 1996) assumed that all cells contain *n* plasmids at division and that plasmids are distributed to daughter cells randomly during cell division, although other models (Nordström and Aagaard-Hansen, 1984) assumed that the probability of replication per unit time is constant throughout the cell cycle and independent of the number of plasmids. In general, theoretical models assume that random segregation of plasmids can be described with a binomial distribution, *B(n,p)*, where *n* is a variable denoting the plasmid copy-number of the mother cell and *p* the probability of success (Greenhalf et al., 1989; Summers, 1991). If we assume there is an equal probability for each plasmid to be inherited to each daughter cell we obtain *p* = 0.5 and, therefore, the probability of producing a plasmid-free cell is 2^(1–n)^.

But plasmid partition is not always symmetric [some plasmids even encode active partition mechanisms that ensure plasmids are segregated symmetrically upon division (Salje, 2010)]. In a recent study (Hsu and Chang, 2019), the authors used fluorescent microscopy to show that plasmids can be localized in spatial clusters inside the cell and therefore segregation of plasmids between daughter cells can be asymmetric (*p≠*0.5). By comparing their experimental results with an impeded segregation model whereby plasmids are spatially confined in the intracellular environment, the authors found that segregation in high copy-number plasmids can deviate from the standard random segregation model.

Other mechanisms can also result in the asymmetric segregation of plasmids (Million-Weaver and Camps, 2014), notably the formation of dimers effectively reduces the units of inheritable plasmids and thus increase the probability of plasmid loss (Nordstrom and Austin, 1989; Paulsson and Ehrenberg, 1998, 2001; Field and Summers, 2011; Werbowy et al., 2017). More recently, Münch et al. (2019) argued that, while low-copy plasmid bacteria exhibit symmetric segregation, in high copy plasmids, one of the daughter cells can receive more plasmids, resulting in unequal segregation. By maximizing the average fitness of the population, the authors used their model to argue that asymmetric segregation of plasmids is an evolutionary stable strategy. Although there is growing evidence that, under certain conditions, segregation of high copy-number plasmids can be asymmetric, modeling plasmid partition as a Poisson process results in a convenient simplifying assumption that enables population dynamic models to describe the segregational loss in terms of a constant rate that depends on the density of plasmid-bearing cells and the mean plasmid copy number (PCN) in the population (Stewart and Levin, 1977; Cooper et al., 1987; Bergstrom et al., 2000; Paulsson and Ehrenberg, 2001; De Gelder et al., 2004; Ponciano et al., 2007; San Millan et al., 2014).

As the probability of segregational loss increases considerably when the number of plasmids carried by each cell is reduced, low-copy plasmids have evolved molecular mechanisms that ensure plasmids are partitioned symmetrically between daughter cells, therefore enhancing their stability in non-selective conditions (Salje, 2010). Of note, active partition mechanisms, whereby plasmid-specific proteins segregate plasmids symmetrically upon division, and post-segregational killing (PSK) mechanisms based on toxin-antitoxin (TA) systems that prevent the emergence of plasmid-free cells through the production of a long-lasting toxin and a shorter-lived antitoxin, both encoded on the plasmid. If the cell carries the plasmid, an antitoxin is produced and the toxin is neutralized, but if a plasmid-free cell emerges in the population, the antitoxin is degraded rapidly and the toxin kills the cell, thus producing a population composed exclusively of plasmid-bearing cells. The evolution of PSK mechanisms appears to be paradoxical because of the detrimental effect they produce on host-cell populations, although theoretical models have shown that PSK can be advantageous for conjugative plasmids (Mongold, 1992) or in the presence of competing genetic elements in spatially structured environments (Mochizuki et al., 2006).

## Modeling Population Dynamics of Plasmid-Bearing Populations

Plasmid replication is a tightly regulated process that ensures enough plasmids are present in the cell at the moment of division [see del Solar and Espinosa (2000) for a comprehensive review on the biology of plasmid replication]. This implies that, at the moment of division, all cells in the population exhibit, on average, a fixed number of plasmid copies. This allows population dynamic models based on ODEs to describe plasmid loss via segregation as a transition between plasmid-bearing and plasmid-free compartments occurring at a rate determined by the mean plasmid copy-number of the population (Cooper et al., 1987; Stephanopoulos and Lapudis, 1988; Bergstrom et al., 2000; San Millan et al., 2014). This is, of course, a simplification, but a convenient one that is compatible with the resolution of experimental protocols designed to estimate mean plasmid copy-numbers in bacterial populations.

Another important feature of mobile genetic elements (MGEs) is that they can impose a physiological burden on the host in the absence of selection for the traits they encode (San Millan and MacLean, 2017). There are multiple mechanisms that have been shown experimentally to produce a fitness reduction to the host, for instance the energetic cost associated with replicating additional DNA (Glick, 1995) or increasing protein synthesis (Rozkov et al., 2004), as well as the production of plasmid gene products that induce genetic conflicts between plasmids and their hosts (Hall et al., 2021) or that inhibit cell division until sufficient plasmid copies are available (Nordström, 1985).

To model the metabolic cost associated with bearing plasmids at a population-level, most population dynamic models express fitness costs in terms of the relative growth difference between plasmid-free and plasmid-bearing sub-populations. For instance, as a constant rate that multiplies the growth function of the sub-population carrying the plasmid by a factor less than one (Simonsen et al., 1990; Baker et al., 2016) or, if bacterial growth is modeled as a hyperbolic function, by appropriately selecting the parameters of the Monod-type growth function (Levin et al., 1979; Stephanopoulos and Lapudis, 1988; Hsu and Tzeng, 2002; San Millan et al., 2014; Alonso-del Valle and León-Sampedro, 2020). Similarly, it could be assumed that, from one generation to the next, the number of plasmid-carrying cells doubles from the previous generation (minus the fraction lost through segregation), so the growth rate of the plasmid-free sub-population can be modeled as 2^1+σ^ where σ denotes the fitness advantage of not carrying the plasmid (De Gelder et al., 2004; Joyce et al., 2005; Ponciano et al., 2007).

Although most population-dynamic models consider plasmid costs to be a fixed value inherent to each plasmid-host association, Ganusov and Brilkov (2002) argued that a constant reduction in growth rate is not realistic when modeling continuous culture devices, as the selection coefficient is a dynamic component of the system. Ponciano et al. (2007) also considered a dynamic plasmid burden by assuming that growth rate of the plasmid-free sub-population is a random variable. This study reported that variability of experimental time-series of plasmid frequencies were captured more accurately by the model with variable fitness-cost than when considering a constant fitness cost throughout the experiment.

### Population Dynamics of Unstable Plasmids

In a seminal study, Stewart and Levin posed a simple ODE model to study the population dynamics of two competing sub-populations: plasmid-free and plasmid-bearing (Stewart and Levin, 1977). The main contribution of this work was to identify a broad range of parametric conditions whereby plasmid-bearing sub-populations can be maintained in the population at high frequencies, even in the absence of selection favoring the genes they carry. The authors derived an expression–now referred to in the literature as the *Stewart-Levin criterion*–that determines the equilibrium frequencies of plasmid-bearing cells in terms of key modeling parameters: population growth, conjugational transfer, and segregation rate. In a follow-up study (Levin and Stewart, 1980), this model was extended to consider non-conjugative plasmids, concluding that the range of conditions that stabilize plasmids are more stringent and that periods of positive selection would be necessary for maintaining costly, non-conjugative plasmids in a population, a result that was later formalized (Macken et al., 1994).

These results were then followed by two theoretical studies based on a system of ODEs that describe changes in the frequency of plasmid-bearing cells from the rate of segregational loss and the selective disadvantage of carrying plasmids. Cooper et al. (1987) showed that the frequency of unstable microbial populations (in this case, plasmid-bearing cells) decreases exponentially in the absence of selection, while Lenski and Bouma (1987) obtained an expression that establishes that the effect of selection is higher when the initial plasmid frequency in the population is low. By comparing their model predictions with an experimentally determined time-series of plasmid frequencies, the authors showed that segregation and selection should be considered simultaneously when addressing the causes of plasmid instability.

Altogether, the contribution of early population dynamics models was to show the existence of important physiological parameters involved in the generation of plasmid-free cells in a population that was composed initially of cells carrying plasmids: (1) the rate at which plasmid-bearers are produced, (2) the rate at which plasmid-free cells are generated through segregational loss, and (3) growth rate differences between both sub-populations. Interestingly, as the burden associated with carrying plasmids depends on the environmental conditions, these studies also showed that it is possible to find selective regimes whereby plasmid-bearing cells grow faster than plasmid-free cells, thus stably maintaining plasmids in the population. However, as mentioned before, there is an apparent paradox associated with frequently beneficial genes: if a plasmid carries genes that confer a benefit for the host, the cell could, in principle, integrate these genes into the chromosome, thus rendering the costly plasmid redundant and unstable in the population.

To study this problem–referred to as the plasmid paradox (Harrison and Brockhurst, 2012)–Bergstrom et al. (2000) postulated a population dynamics model and used it to show that plasmids cannot persist exclusively by carrying genes that are beneficial to their hosts. The main result of this study was to show that plasmids can only persist indefinitely through a series of selective sweeps resulting from transferring to locally adapted populations with higher fitness, a process referred to as the “*hitchhiking hypothesis*.” However, a detailed analysis of an extension of this model that considers resource limitation by modeling carrying capacity as an explicit parameter, showed that the conditions that enable a parasitic plasmid to persist are less stringent than originally thought, and that plasmids can be maintained indefinitely through oscillations between plasmid-free and plasmid-bearing populations (Lili et al., 2007). These complex dynamics result from considering a variable population size, in contrast to population genetic models that generally assume a finite and constant population size. In this case, plasmids are inevitably driven to extinction (or fixation) in the absence of immigration (Novozhilov et al., 2005; Tazzyman and Bonhoeffer, 2013).

### Plasmid Stability in Fluctuating Environments

Most plasmid population dynamics studies discussed so far describe resource limitation implicitly in the model (e.g., by considering growth rate as a logistic function with a limited carrying capacity). In turn, Stephanopoulos and Lapudis (1988) included a term representing substrate concentration in the environment, allowing the authors to demonstrate the existence of a range of substrate concentrations where both sub-populations co-exist in the long-term. But not all environmental conditions maintain plasmids indefinitely. Indeed, as stated by the Stewart and Levin criterion, a necessary condition for plasmid persistence is that the rate of horizontal transmission should compensate for the fitness cost associated with plasmid bearing. An exhaustive analysis of genomic sequences, however, showed that a large fraction of plasmids found in nature is not mobilizable (Smillie et al., 2010), suggesting the existence of other extrinsic and intrinsic factors driving plasmid persistence.

A possibility is that constant selection in favor of plasmid-encoded traits is not a necessary condition for plasmid maintenance, and that sporadic intervals of positive selection would be sufficient to rescue plasmids from extinction. In this respect, mathematical modeling has been used to study plasmid dynamics in response to different selection intensities and intervals between treatments (Svara and Rankin, 2011). Another study analyzed a competition model growing in a chemostat in the presence of an antimicrobial substance deployed in periodic pulses, showing that the dilution rate and the periodicity of drug input are critical for the stable coexistence of both sub-populations (Yuan et al., 2011). More recently, Rodriguez-Beltran et al. (2018) used stochastic simulations to show that a high rate of environmental fluctuations was correlated with an increase of genetic diversity in the population, resulting in an enhanced plasmid stability.

## Co-Evolution Between Bacteria and Their Plasmids

As plasmids encode for genes that control their replication and transmission, it has been argued that they should be viewed as evolving agents subject to natural selection in their own right, with fitness interests that are not necessarily aligned with those of their bacterial host (Harrison and Brockhurst, 2012). As a result, the evolutionary dynamics of plasmid-host associations can be understood as a co-evolutionary process, where both conflict and collaboration between bacteria and plasmids can occur.

### Plasmid Copy-Number as an Evolvable Trait

An effective strategy to stabilize plasmids in a population is to reduce the probability of plasmid loss by compensating plasmid costs or by reducing segregational loss (e.g., through plasmid addiction systems or active partition mechanisms). But not all plasmids encode such molecular mechanisms, so an alternative strategy to mitigate segregational loss is to increase the number of plasmid copies carried in each cell. Besides increased stability, there are other potential benefits associated with an increase in copy-number (e.g., increase in gene dosage, or producing genetic redundancy that enables different versions of plasmid-encoded genes to transiently co-exist in the same cell).

However, large numbers of plasmids can also be detrimental as they produce a significant reduction in the growth rate of their bacterial hosts. This trade-off between fitness cost and plasmid stability has been shown, both *in vitro* and in theory, to result in the existence of an optimal plasmid copy-number susceptible to be tuned by evolution, with strong selection against increasing the number of plasmids carried in each cell (Mei et al., 2019). A modeling study showed that selection against extreme copy-number plasmids can produce ecological cycles between mutants with low, intermediate, and high copy numbers, an eco-evolutionary process that can theoretically increase plasmid stability (Watve et al., 2010). Another important aspect of plasmid evolutionary dynamics is the interaction between plasmid copy-number and evolvability. For instance, in a plasmid-encoded gene, the mutational target size is amplified by the number of copies of the plasmid, therefore producing a positive correlation between plasmid copy-number and the probability of mutation (San Millan et al., 2016; Dimitriu et al., 2020).

The complex interaction between plasmid copy-number and random genetic drift was studied by Ilhan et al. (2019), using a combination of experimental evolution and a population genetics model based on a standard haploid version of the Wright-Fisher model that incorporates plasmid evolution following an approach previously used to study mitochondrial evolution (Peng and Kimmel, 2005). By performing forward-time simulations of the model with constant population size, this study estimated allele frequencies over time in terms of the selection coefficient in favor of mutant alleles, the initial mutant frequency, and random genetic drift. Moreover, by evaluating the stability and evolvability of plasmids with different PCNs, the authors showed that mutations on plasmid-encoded genes are susceptible to be cleared from the population through segregational drift. In consequence, the frequency of mutations occurring in plasmids is reduced compared to mutations encoded in the chromosome, and the time to fixation for high copy-number plasmids increases, results that were confirmed using experimental evolution and genome sequencing.

### Fitness Cost Compensation

Besides tuning PCN so that the risk of segregational loss and the cost associated with increasing plasmid content is balanced, theoretical studies have also shown that other evolutionary routes increase plasmid stability in the long term. Notably, compensatory evolution that reduces the cost of plasmid carriage and therefore weakens selection against plasmids has been shown, both theoretically and experimentally, to increase plasmid stability. For instance, Harrison et al. (2016) simulated the evolutionary dynamics of a very costly mega-plasmid with a mercury resistance cassette carried on a transposon using a computational model that explicitly considered the appearance of compensatory mutations and the transposition of resistance genes into the chromosome. Computer simulations of the model showed that plasmid cost compensation enhances plasmid stability and promotes the fixation of accessory traits on the bacterial chromosome. In a follow-up study, the authors extended this model to show that, due to the inherent costs of increasing the rate of horizontal transmission, amelioration of plasmid costs appears to be the most likely long term solution to evolve stable bacteria-plasmid associations (Hall et al., 2017).

This result is consistent with another study evaluating the effect of compensatory mutations in the stability of a costly, non-conjugative plasmid (San Millan et al., 2014). In this study, the authors performed a stability assay in the absence of selection and found that, after an initial period of exponential decay, the plasmid appeared to be stabilized at low frequencies. This result was at odds with a simple population dynamic model that predicted the plasmid-bearing population would be driven to extinction. This apparent discrepancy between theory and data provides an opportunity, both to theorists and experimentalists, to perform further analysis and gain insight into the underlying process. In this case, the authors found that extending the population dynamics model to consider a subpopulation with a reduced fitness cost resulted in stability patterns similar to those observed *in vitro*. Indeed, whole genome sequencing and competition experiments between the evolved and ancestral strains showed that the increased stability observed in the long term was a consequence of a substantial reduction in fitness cost associated with plasmid-bearing in the evolved strains.

To evaluate the consequences of genome localization of compensatory mutations in the resulting plasmid dynamics, Zwanzig et al. (2019) postulated and analyzed an ODE model to argue that compensatory mutations occurring in plasmids (spreading both vertically and horizontally) can promote plasmid persistence even when the amelioration effect is less compared to the produced by chromosomally encoded compensatory mutations that are only inherited vertically. As with most of the studies discussed so far, the goal of this manuscript is to use mathematical modeling to obtain a qualitative understanding of the behavior of a complex system under a wide range of conditions and parameter values, with model predictions that can then be compared with data obtained experimentally.

An alternative approach is to compute the maximum-likelihood estimates of different parameters of a mathematical model from experimental data, with the aim of making inferences about the underlying biological processes that generated the patterns observed *in vitro*. This approach was used by De Gelder et al. (2007), to fit plasmid persistence curves under non-selective conditions and parametrize a plasmid population dynamics model published previously (De Gelder et al., 2004; Ponciano et al., 2007). Interestingly, experimental data revealed a great diversity of generation times to plasmid loss and, by considering different replication and segregational loss rates in the model, the authors concluded that this variation could be due to plasmid re-uptake, but most likely from compensatory mutations that result in a variable plasmid cost.

In a follow-up study (De Gelder et al., 2008), the authors isolated the two strains for which the plasmid was less stable and evaluated plasmid adaptation using experimental evolution, either in a single species or when alternating hosts. Parameter estimation of the model suggested that, besides segregational loss and plasmid costs, other strain-specific factors appear to affect the stability dynamics at an evolutionary time-scale, notably compensatory mutations that reduce plasmid cost over time. This approach was also used to evaluate the contribution of different model parameters (fitness costs and segregational loss) in the host range expansion observed experimentally (Loftie-Eaton et al., 2016). Similarly, by jointly estimating different parameters (conjugation rate, segregation rate, and plasmid cost) and analyzing data from competition experiments and plasmid persistence assays, Loftie-Eaton et al. (2017) concluded that plasmid maintenance was improved by the appearance of chromosomal mutations that turned a plasmid cost into a benefit, a theoretical result that was subsequently confirmed by sequencing the evolved strains.

## Modeling Horizontal Transmission of Plasmids

We have discussed a series of theoretical studies showing that intermittent intervals of positive selection result in enhanced stability of plasmids. But selection itself is not sufficient to explain the abundance of plasmids in natural environments. For instance, as an extreme case, cryptic plasmids are ubiquitous in nature, although they carry no beneficial genes and thus are never positively selected for. So, another strategy to increase plasmid stability is to overcome the negative demographic effects of segregation and purifying selection by transmitting horizontally into neighboring plasmid-free hosts (Simonsen, 1991). As HGT enables the dissemination of antibiotic resistance genes, this problem has received considerable attention, both from clinicians (Carattoli, 2013) and from mathematical modelers (Leclerc et al., 2019).

Horizontal transmission of MGEs is mainly mediated either via conjugation, transformation, or transduction (Smillie et al., 2010), and therefore plasmids can be classified as conjugative (when encoding mechanisms for self-transfer) or non-conjugative (if they are incapable of initiating conjugation for self-transmission). As with other forms of HGT, conjugation has been associated with promoting evolutionary and ecological innovation, by conferring new phenotypic traits and access to novel ecological niches (Wiedenbeck and Cohan, 2011), although it can also be detrimental when selfishly replicating MGEs can spread in the population in the absence of selection and does not encode for beneficial genes (Vogan and Higgs, 2011).

A recent study used a Wright-Fisher type model to argue that parasitism (whereby a costly plasmid is maintained exclusively through transmission) is a viable plasmid lifestyle, as it enables plasmids to establish in the population when transmission rates are high and selection levels are low (Lopez et al., 2021). The authors also note that, although parasitic plasmids could, in theory, spread in the population until all cells carry the runaway plasmid, this is not what is observed *in vivo*. By extending their model to consider plasmid co-infection, the authors demonstrate that the reduction in fitness imposed by the parasitic plasmid results in a higher probability of being out-competed by populations with fewer plasmids.

Crucially, conjugation can occur between taxonomically distinct bacterial lineages (Fernandez-Lopez et al., 2017), so phylogenetic relationships can be confounded and beneficial genes can spread in the population rapidly between members of multi-species microbial communities. The dissemination of plasmids in microbial populations is therefore an important problem that has been extensively studied in theory, mostly using ODEs when evaluating HGT in well-mixed environments or PDEs when considering the spatial component of the system. As PDEs are difficult to analyze mathematically, other approaches have also been used to study plasmid transfer in spatially explicit environments, notably lattice-based computational models. In this section, we will discuss different frameworks used to study the horizontal transmission of plasmids.

### Mass Action Assumption

It has been established that the dynamics of horizontal transfer differ between populations on surfaces and in liquids (Molin and Tolker-Nielsen, 2003) but, as most articles studying theoretical population dynamics of plasmids focus on either chemostats or batch culture experiments (i.e., dense cultures in well-mixed environments), considerit is usually considered that conjugative plasmid transfer between donor and recipient subpopulations can be modeled based on mass-action kinetics. In consequence, a simplifying assumption of most population dynamic models is that transitions between donor and recipient compartments are proportional to their respective densities, an assumption that results from considering that conjugation events occur at a rate depending on the conjugative efficacy and permissiveness of both donors and recipient cells (Stewart and Levin, 1977; Freter et al., 1983; Clewlow et al., 1990; Simonsen et al., 1990; Macdonald et al., 1992; Smets et al., 1993; Bergstrom et al., 2000; Imran et al., 2005; Svara and Rankin, 2011; Peña-Miller et al., 2015; Lehtinen et al., 2021).

However, experimental studies have shown that the rate of conjugation is not constant but can depend on resource availability (Philipsen et al., 2010)quorum-sensing signals (Miller and Bassler, 2001; van Gestel et al., 2021) and the presence of antimicrobial substances (Beaber et al., 2004). Indeed, by fitting a mass-action model to experimental data, Levin et al. (1979) observed plasmid transmission accelerated during lag phase, and therefore argued that considering a constant rate of transmission is only appropriate during stationary phase. In another study, Lundquist and Levin (1986) showed that newly formed transconjugants can transiently promote conjugative pili synthesis, resulting in an increased rate of horizontal transfer relative to the original donor cell. In contrast, Simonsen (1991) argued that the initial donor/recipient ratio and the occurrence of a lag phase have no appreciable influence in the estimation of transfer rates, thus arguing that the rate of horizontal transmission remains constant throughout the experiment.

The interaction between the intensity of positive selection with the rate of environmental change and horizontal transmission was studied numerically, concluding that conditions for the maintenance of non-transmissible plasmids in drug-free environments can be very stringent (Peña-Miller et al., 2015). In consequence, the authors argue that, in natural environments, non-transmissible plasmids may still experience rare episodes of horizontal transmission that stabilize parasitic plasmids in bacterial populations. For conjugative plasmids, Lopatkin et al. (2017) showed that plasmids transfer at sufficiently high rates to be maintained in the absence of antibiotics, thus concluding that reducing antibiotic use alone would not reverse resistance from the population.

For conjugation to occur, plasmid-bearing and plasmid-free cells first need to collide, attach, and then successfully conjugate before detachment occurs. The mass-action kinetic assumption of most mathematical models of plasmid transfer combines these processes into a single conjugation rate, implicitly ignoring the physiological state of donor and recipient cells and considering that horizontal transmission of plasmids is an instantaneous process. To overcome these limitations, a model for conjugative plasmids that included time lags was proposed (Massoudieh et al., 2007), with delays resulting from reversible attachment–detachment of bacteria in a one−dimensional porous environment. Similarly, Zhong et al. (2010) postulated an ODE model that explicitly considered attachment and detachment dynamics of neighboring cells, assuming that conjugation can only occur between attached cells. This assumption allowed the authors to explore the differences in the dynamics of plasmid transfer between spatially structured and well-mixed environments, arguing that vigorous shaking negatively affects plasmid transfer and that plasmid transfer in liquid environments is optimized at low to moderate shaking speeds.

Although deterministic models have been very useful to study the spread and persistence of conjugative plasmids in well-mixed environments (e.g., liquid cultures), the implicit limitations resulting from using mean-field approximations limit the study of the stochastic interactions between plasmid-bearing and plasmid-free cells. A stochastic model that assumes mass-action kinetics was postulated (Novozhilov et al., 2005), and used to show that horizontally acquired plasmids can be maintained for long intervals of time in populations when horizontal transmission is comparable to segregational loss, even when the acquired genes are neutral or deleterious. Other stochastic modeling approaches have also been used to study HGT. For instance, Tazzyman and Bonhoeffer (2013) studied the fixation probability of a gene that can be horizontally transferred using two modeling approaches: a branching process and a diffusion approximation. By considering a fixed population size and a low initial frequency of mutants, these models showed the existence of a trade-off between horizontal and vertical transmission that results in deleterious alleles having a non-zero probability of fixation, even in the absence of positive selection.

### Plasmid Transmission in Space and Time

As plasmid transfer by conjugation requires physical contact between donor and recipient cells, spatial structure plays an especially important role in the dynamics of HGT [see Slater et al. (2008) for a review on spatial factors modulating plasmid transmission]. However, most studies focus on well-mixed environments and ignore the spatial component of the system. To relax this assumption, Beaudoin et al. (1998) posed a model based on PDEs to study plasmid mobilization in a 1-D biofilm (spatial structure is expressed in terms of the depth of the biofilm). In principle, this PDE approach could be extended to two- or three-dimensional domains, although it would be very difficult to analyze. For this reason, computational models have been used by multiple studies to simulate the conjugative transfer of plasmids in spatially explicit environments.

In short, IBMs assume that each individual is described by a vector of state variables that is updated in response to rules based on its current state, the state of neighboring cells, and local environmental conditions. This framework has been used successfully to study competitive and cooperative microbial interactions in space and time (Hellweger et al., 2016) and has been argued to be a powerful approach to study plasmid transmission, incompatibility, and host range in biofilms and other types of surfaces, as well as to study plasmid-encoded antibiotic resistance (Kreft, 2014). In the context of HGT, Lagido et al. (2003) proposed a simple kinetic model of plasmid transfer within microcolonies growing in solid surfaces, while Zwanzig (2021) incorporated genetic and metabolic information about individual cells and used computer simulations to evaluate population-level behavior. This computer-intensive approach has also been used to study the effect of environmental heterogeneities (e.g., nutrient and antibiotic gradients) in the spatio-temporal dynamics of HGT, for instance by considering a grid-based environment where abiotic substances interact locally with bacterial cells and diffuse between neighboring microenvironments (Gregory et al., 2006, 2008).

Another individual-based approach used to study plasmid transfer and persistence in spatially structured bacterial populations is based on discrete-space continuous-time stochastic models (also known as interacting particle systems). Of note, Krone et al. (2007) used a 2-D square lattice with periodic boundaries, whereby each location of the lattice can contain nutrients and recipient, donor, or transconjugant cells. A similar IBM that considers cells, plasmids, and extracellular polymeric substances was used to study plasmid transmission in biofilms (Merkey et al., 2011) and, by performing sensitivity analysis, showed that timing and distance between neighbors are important drivers of conjugal plasmid transfer in biofilms, while segregational loss rate and growth rate of the receiver sub-population appear to be less relevant.

More recently, Werisch et al. (2017) evaluated the difference between non-transmissible and transmissible plasmids in biofilms modeled using a lattice-based IBM. In this computational model, conjugation occurs randomly between neighboring cells, while segregational loss and incompatibility occur when both types of plasmids are present in the same cell. The main conclusion of this study is that non-transmissible plasmids that provide no advantage to the host can still be maintained in the population in co-occurrence with incompatible conjugative plasmids. In turn, van Dijk et al. (2020) used an IBM to study a population of bacterial cells carrying (or not) a slightly beneficial gene to show that HGT can only evolve if horizontal transmission occurs within spatially localized populations instead of under well-mixed environmental conditions.

## Modeling Plasmid-Host Associations: Which Genes Are Carried on Plasmids?

Interestingly, while certain genes encoding for specific functions (e.g., catabolism, resistance, virulence, and interference competition) are usually present on plasmids rather than chromosomes, others (such as genes involved in transcription or translation) are usually chromosomally encoded. Several hypotheses have been postulated to explain the over-representation of certain characters encoded in plasmids, with their causes and implications analyzed using mathematical models. For instance, the local adaptation hypothesis states that many of the characters that tend to be present on plasmids are adaptations to local variations in environmental conditions that occur only sporadically in time or in space (i.e., genes are only useful in certain environments or at certain times). This theory was proposed by Eberhard (1990) and is based on the observation that sometimes-useful genes linked to horizontally transmissible elements out-compete non-mobile versions of the same genes by associating with bacterial genotypes with increased fitness. More recently, Tazzyman and Bonhoeffer (2014) used a stochastic birth-death model to show that plasmids are not necessarily beneficial locations for resistance genes. In a follow-up study (Tazzyman and Bonhoeffer, 2015), the authors used a population dynamics model that compares degradation rates (loss of function by mutation, deletion, or translocation) for chromosomally encoded and plasmid-encoded genes, concluding that carrying essential genes in plasmids is only beneficial when chromosomal degradation is fast and plasmid cost is low.

A recent study (van Dijk et al., 2020) used an ODE model of a bacterial population undergoing uptake of genes from a shared DNA pool to evaluate the benefit of HGT based on distinct gene classes of slightly beneficial genes: indispensable (where HGT is not required and does not provide a benefit for the host), enrichable (maintained without HGT, although with increased fitness when acquired horizontally), rescuable (cleared from the population without HGT), unrescuable (also maintained only through horizontal transmission, but presenting reduced fitness in the presence of HGT), and selfish elements (only persisting at high rates of HGT and always decreasing growth of the population). Based on this classification, the authors showed that HGT can be an evolutionarily stable strategy for enrichable and rescuable genes, although the absence of gene-carrying donor cells renders HGT evolutionary inaccessible for rescuable genes. Moreover, using an IBM, the authors showed that spatial structure constrains the maintenance of slightly beneficial genes and that, once stable communities have evolved, selfish genetic elements can be stably maintained in the population.

The repertoire of genes carried in plasmids also depends on their degree of dominance, as discussed in a recent manuscript (Rodriguez-Beltran et al., 2020). By combining experiments with stochastic simulations of a fluctuation assay, the authors found a positive correlation between PCN and the frequency of phenotypic mutants for mutations of high dominance, and a negative association for mutations of low dominance. Both the model and the experimental data presented in this study conclude that the repertoire of genes carried in plasmids is determined by the degree of dominance of the genes it carries. Interestingly, the degree of dominance of plasmid-carried alleles has been shown to depend on gene dosage and the environment, with important consequences for the adaptive evolution of bacterial populations carrying non-transmissible multicopy plasmids (Santer and Uecker, 2020). Finally, Ledda and Ferretti (2014) proposed a model for plasmid fitness depending on its length, arguing that the use of antibiotics can increase both the length of plasmids and the number of antibiotic resistance genes carried by each plasmid.

### The Role of Plasmids in Social Evolution

Plasmids have been shown to carry a disproportionate number of genes involved in bacterial virulence and cooperation, suggesting a key role for plasmids in bacterial social evolution. This hypothesis was explored by using a within-host mathematical model to argue that mobility is beneficial because it enforces cooperation between neighboring cells (Smith, 2001). As secreted proteins are costly to produce (the authors focus on virulence factors, but this argument could also be applied to nitrogen fixation, micro-environment detoxification, and other proteins secreted to the extracellular environment), then a microbial community would be susceptible to the invasion of cheaters that fail to produce the public good (and therefore avoid the metabolic cost associated with the production), but still, obtain the benefit resulting from other members of the community producing and exporting the public good. As a result, cheaters would increase in frequency and render the cooperative behavior unstable. This observation highlights a fundamental problem in sociobiology: how can cooperation persist? Theoretical studies have suggested that plasmids can stabilize cooperative behavior, either forcing cheaters to produce the public good by horizontally transmitting a plasmid-encoded cooperative gene (Nogueira et al., 2009) or through the evolution of PCN control in non-conjugative plasmids (Kentzoglanakis et al., 2013).

Nogueira et al. (2009) showed that, when the production of public goods is costly, HGT via plasmids increases local relatedness by infecting previously unrelated neighbors, therefore promoting cooperation. The main result of this study is that the invasion of cheaters in a population of cooperators could be prevented if the social trait was encoded in a conjugative plasmid, through the re-acquisition of the cooperative trait. As relatedness increases through horizontal transmission, the authors suggest that, in theory, cooperative traits (e.g., secreted and outer membrane proteins) should be preferably encoded in mobilizable regions of genomes (Rankin et al., 2011). This result was contended in a following manuscript (Giraud and Shykoff, 2011), arguing that local transmission of uninfected cells is enough to maintain the production of public goods, without the need for invoking kin selection.

The interaction between relatedness (measured at the locus of interest) and HGT has been a subject of controversy and extensive research. Indeed, when a plasmid encodes for cooperative genes, there is the potential for conflict between the plasmid and the host chromosome. Mc Ginty and Rankin (2012) showed that this conflict can be resolved either by controlling the expression of plasmid-encoded genes via a chromosomal suppressor or by exhibiting complete resistance to the plasmid. In a follow-up paper, the authors used a population genetics approach to show the existence of a positive feedback between transmission and relatedness; if individuals are less related in a patch, there would be more available cells to infect, resulting in an increase in overall transmission and therefore increasing relatedness (Mc Ginty et al., 2013).

Importantly, while HGT can favor the initial invasion of cooperation, it is not clear if it favors the long-term maintenance of cooperation (Mc Ginty and Rankin, 2012). In a recent study combining bioinformatic analysis with mathematical modeling, it was argued that, as an invasive plasmid spreads in the population, the plasmid-bearing subpopulation increases in frequency, thus the benefits of HGT decrease in time. As a consequence, the population becomes susceptible to being invaded by non-producing plasmids and the cooperative interaction becomes unstable. Moreover, the authors showed that the resulting population dynamics is analogous to the case where the cooperative gene is encoded in the chromosome, thus rejecting the hypothesis that HGT promotes cooperation (Dewar et al., 2021).

Plasmid-mediated antibiotic resistance can also promote collective resistance in the community, as drug-degrading enzymes released to the environment provide cross-protection to susceptible, plasmid-free cells. Yurtsev et al. (2013) postulated a simple population-dynamics model to show that the cooperative nature of antibiotic inactivation enables co-existence between sensitive and resistant cells, even in the absence of spatial structure. More recently, a competition model between resistant and sensitive bacteria (with resistance carried either in a plasmid or in the chromosome) was used to show the existence of positive frequency-dependent selection on gene location (Lehtinen et al., 2021), a property that emerges from considering that having both chromosomal and plasmid-borne copies of the gene provides no additional benefit than carrying a single copy of the gene (i.e., the increase in resistance resulting from bearing a second copy of the gene is less than the cost of carrying it).

## Modeling Plasmid Dynamics in Natural Environments

Most of the theoretical studies discussed so far have focused on analyzing plasmid dynamics on novel host–plasmid combinations under controlled laboratory conditions, whereas in nature HGT occurs in much more complex environmental and community contexts, for instance in the soil or the mammalian gut. However, studying MGEs in multi-species microbial communities is a difficult problem that spans multiple levels of complexity and presents intrinsic limitations associated with obtaining data (most microbial species cannot even be grown in laboratory conditions). Another difficulty arises when trying to estimate prokaryotic diversity in natural microbial populations or to infer phylogenetic relationships between species, with plasmids at the core of this complexity by obscuring taxonomic classifications and diffusing boundaries between bacterial species (Doolittle and Zhaxybayeva, 2009).

The difficulties inherent in observing microbial communities in natural settings have hindered the application of ecological theory developed in the context of plants and higher animals to study microorganisms (Prosser et al., 2007). In this section, we will discuss studies that extend simple population dynamic models to study plasmid transmission in complex ecological settings. We will also present other studies that evaluated plasmid transfer in multi-strain communities co-existing in controlled laboratory conditions. Altogether, these studies highlight the importance of population diversity in the dynamics of plasmid transmission, in particular, variability in plasmid fitness effects in different hosts and heterogeneity in inter-species plasmid transfer. Finally, we will also briefly discuss epidemiological models used to describe the spread of plasmid-encoded antibiotic resistance genes between individuals. These models can be combined with high-throughput data to study the horizontal transmission of plasmids and, with the aid of statistical methods, study the dissemination routes of drug-resistance genes in clinical settings.

### Plasmid Transfer in the Gut Microbiota

An important issue driving plasmid-mediated dissemination of antibiotic resistance is that commensal bacteria can harbor resistance genes that can potentially be transferred to pathogenic bacteria. A theoretical study focusing on the role of antibiotic resistance in the microbiome was published by Tepekule et al. (2019). This model consists of a system of ODEs that describes the rate of change of different species over time and was used to argue that treatment history has a significant impact on the prevalence of resistance.

A discrete-time mathematical model in the presence (or absence) of a natural gut microbial community was used to evaluate how antibiotic resistance is influenced by the presence of other species (Klümper et al., 2019). In particular, this study evaluated the interaction between *Escherichia coli* and a pig-gut microbiota and showed that selection against resistance occurs at higher antibiotic concentrations when in the presence of other strains. Similarly, other studies have postulated a dynamical model of plasmid transfer in a mouse intestine (Freter et al., 1983), and studied the effect of different antibiotic regimes and composition of the gut microbiota in the levels of resistance observed in a pig gut (Ahmad et al., 2015).

While most studies focusing on the kinetics of plasmid transfer in the animal gut consider that the intestine can be approximated by a continuous-culture device (ignoring the spatial structure and assuming perfect mixing of donor and recipient cells), either by considering compartmentalization of the intestinal environment (Licht et al., 1999) or by evaluated the environmental dynamics of a gut colonized by two bacterial populations, each carrying a non-conjugative plasmid with a TA system, competing in a spatially extended habitat referred to as a flow reactor (Grover and Wang, 2019). Using numerical simulations of a system of PDEs, the authors explored the conditions that allowed for co-existence between plasmid-bearing and plasmid-free sub-populations, concluding that segregation rate and fitness cost of plasmid carriage must be relatively low for both strains to co-exist.

### Plasmid Transmission in the Environment

It is well established the environment can be a source of resistance genes that can be transferred into clinically relevant bacteria (Stotzky and Babich, 1986). In this respect, mathematical modeling has been used to study transmission and maintenance of plasmids in bacterial populations living in the gastrointestinal tract of livestock or their associated farm environment, as well as to perform risk assessments on genetically engineered organisms released into the environment (Weinberg and Stozky, 1972; Graham and Istock, 1978; Landis et al., 2000). Pharmacokinetic–pharmacodynamic models have also been used to study the dissemination of antimicrobial resistance genes at the farm level (Lanzas et al., 2011) and to estimate the length of a drug withdrawal period before slaughter, to reduce resistance levels before meat consumption (Cazer et al., 2018).

Another study used a simple mathematical model to evaluate the spread and selection of antimicrobial-resistant bacteria in a slurry tank of a dairy farm (Baker et al., 2016). This model is based on a previous study by Volkova et al. (2012), and uses ODEs to describe the growth of each subpopulation and the inflow of fresh slurry containing both sensitive and resistant bacteria, as well as gene transfer and selection for plasmid-encoded resistance genes. By performing global sensitivity analysis and numerical simulations, the authors showed that the rate of horizontal transmission and the length of time that slurry is stored in the slurry tank (without outflow and thus considering the tank increases in volume) are the most significant parameters driving plasmid maintenance and therefore good targets for preventing antimicrobial-resistant pathogens entering the human food and water supply chains.

### Plasmid Dynamics in Polymicrobial Synthetic Communities

To overcome the complexities associated with studying plasmid ecology and evolution in natural environments, a few studies have used *in vitro* model systems consisting of synthetic communities composed of multiple species co-existing in controlled environmental conditions. For instance, Gama et al. (2020) extended previous models (Stewart and Levin, 1977; Levin et al., 1979; Simonsen, 1991) to incorporate multiple interacting plasmids, with the aim of evaluating how the interaction between plasmids impacts plasmid persistence in bacterial communities. This study postulates a hierarchy in the processes relevant for plasmid maintenance, and uses mathematical modeling to show that epistatic interactions between plasmids produce a stronger impact than other parameters influencing conjugation and segregational loss.

The co-evolution of host-plasmid pairs in a mixed-species culture of *Klebsiella pneumoniae* and *E. coli* was studied theoretically and experimentally by Jordt et al. (2020), showing that pleiotropy enhances plasmid stability in microbial populations when the plasmid had previously co-evolved with one of the members of the community. Similarly, Alonso-Del Valle et al. (2021) studied the distribution of fitness effects of a clinically relevant plasmid in different *Klebsiella* and *E. coli* isolates obtained from clinical samples. Using a population-dynamics model, the authors showed that variability in the cost of plasmid-bearing between different hosts can stabilize plasmids in polymicrobial communities and reduce the critical conjugation rate necessary to stabilize plasmids. Moreover, using computer simulations, this study showed that plasmid frequency is a decreasing function of community complexity when plasmid-bearing is associated with a constant fitness cost, but an increasing function of the number strains in the community when the distribution of fitness effects presents a large variance.

A series of recent articles have used theoretical models to study the invasion of multiple plasmids in mixed populations. Of note, Lehtinen et al. (2021) postulated a stochastic model of resistance acquisition and transfer in a bacterial community that considered that a beneficial gene is transferable between *n* possible bacterial species, either through transformation (horizontal transfer between species of chromosomal resistance) or through conjugation (resistance transfer between species). The main result of this study is that the probability of finding genes on plasmids increases with higher rates of inter-species plasmid transfer and with a higher number of species between which the gene can be shared. The consequence of this frequency dependence is that moderately beneficial genes can be maintained on plasmids, despite segregation loss, if they are present at a higher frequency.

Also recently, Wang and You (2020) proposed a plasmid-centric framework to analyze gene flow in complex communities based on the overall abundances of *m* plasmids spreading in a community composed of *n* bacterial strains. This study also proposes a heuristic expression based on growth kinetic parameters of individual strains to estimate the persistence potential of plasmids in multispecies microbial communities. Lopez et al. (2021) used a multiplasmid and multipopulation Wright–Fisher type model to argue that HGT barriers determine the distribution of unique plasmid types across populations.

An aspect that is usually overlooked is the influence of viruses in the ecology and evolution of plasmid-bearing populations. Dionisio (2005) proposed a chemostat model whereby male-specific phages that can only infect donor cells and showed that heterogeneity in the rate of transfer is a critical parameter influencing the stability of conjugative plasmids in bacterial communities when conjugative plasmid–dependent phages are present. Also, by combining experimental observations with mathematical modeling and computer simulations of an IBM, Harrison et al. (2015) studied the interaction between lytic bacteriophages and the persistence of conjugative plasmids and showed that the population is under strong indirect selection pressure from lytic bacteriophages, therefore limiting the ecological conditions where plasmids can persist.

### Epidemiology of Plasmids

The epidemiological dynamics of infectious diseases can be described mathematically, either using within-host models to study the evolution of resistance within a treated host or between-hosts models to describe the spread of resistance in a community of hosts (Blanquart, 2019). Between-host epidemiological models of antibiotic resistance are usually based on simple compartmental models whereby individuals can be classified as susceptible, recovered, or colonized by drug-sensitive or drug-resistant bacteria. Transitions between these compartments results from transmission events or as a consequence of drug treatment. Only a few epidemiological studies have explicitly focused on plasmid-borne resistance, although several have studied plasmid dynamics implicitly by including HGT in epidemiological models of drug resistance. For instance, Levin et al. (2014) postulated an epidemiological model to argue that the frequency of resistance is maintained due to the presence of plasmids, although it can also be reduced by decreasing antibiotic use, constraining the development of resistance during treatment, or by restricting invasion of resistant pathogens into the community.

Recent studies have also combined high-throughput data and statistical models to study dissemination routes in hospital settings. Leon-Sampedro et al. (2020) analyzed epidemiological data from 9,000 patients and whole-genome sequencing data from 250 enterobacteria clones to study the spread of a carbapenemase-encoding plasmid (pOXA-48) in a hospital over 2 years. A model selection approach based on a case-specific probabilistic model that implements a Markov chain Monte Carlo algorithm was used to make inferences about the dissemination routes observed in the data. The source of the outbreak was identified using a structured coalescent-based tool for reconstructing bacterial transmission. pOXA-48 also produced a hospital outbreak of carbapenem-resistant Enterobacteriales in the United Kingdom, which was studied by Ledda et al. (2020) with a mathematical model for conjugation (modeled as a homogeneous Markov process between bacterial hosts), allowing the authors to identify the founder strain responsible for the outbreak and to calculate the number of conjugation events that occurred during the outbreak.

Although the aforementioned studies focus on localized outbreaks, more general models have also been proposed to study plasmid-driven spread of antibiotic resistance genes at an epidemiological level. For instance, through a computer-intensive approach that implements a multi-scale individual-based system where individuals are confined within abstract structures referred to as “membranes,” which in turn are organized into tree-like structures. Campos et al. (2019) used this membrane computing approach to study microbial evolution at multiple complexity scales: genes, phenotypes, cells, populations, communities, and ecosystems. Using this methodology, the authors studied the multihierarchical processes involved in antibiotic resistance by introducing a resistance plasmid and a conjugative element (which transfers a resistance gene into the chromosome) into a hospital setting. This study concludes that, in the long term, the dissemination of resistance genes would be more effective when they are encoded in plasmids instead of in the chromosome.

## Discussion

The wide range of modeling approaches discussed in this review can be classified according to different criteria, for instance, based on whether the spatial structure is considered, or if the environment is assumed to be homogeneous. The latter enables us to assume mass action kinetics and therefore changes in plasmid frequency can be described using ODEs, while the former is usually studied either using PDEs or IBMs. Another classification is based on how models deal with randomness and uncertainty. In deterministic models, the output of the model is fully determined by the parameter values and the initial conditions and is usually based on differential equations where the state of the system is continuous and therefore is expressed in terms of concentrations and cell densities, instead of molecules and cell numbers. In microbial population biology, this assumption has been shown to hold when considering a dense culture growing in a well-mixed environment. In this case, the state of the system can be represented with a vector containing the densities of different sub-populations and its temporal dynamics described using a system of deterministic differential equations.

But noise is an inherent property of biological systems and particularly so in plasmid biology. Indeed, in this review we have discussed several sources of noise generated by different aspects of plasmid lifestyle: replication occurs in discrete events stochastically distributed in time, multicopy plasmid partition is an inherently noisy process, and random collision and attachment of donor and recipient cells is a necessary condition for plasmid conjugation. Moreover, the environment can also be variable, as well as the fitness costs associated with each plasmid-host association, both resulting in a wide distribution of plasmid fitness effects. To explicitly consider noise, mathematical models can be postulated such that probability distributions of potential outcomes are estimated by allowing for random variation in one or more inputs over time.

In general, stochastic models are more difficult to analyze, mainly because the analytical solution of stochastic equations is usually intractable if a large number of interactions are involved. However, the increase in computational power and the development of numerical algorithms in the past decades provide mathematical modelers the possibility of obtaining trajectory ensembles of stochastic models with statistics that asymptotically converge to the solution of the corresponding deterministic equation. This simulation-based approach, however, also has its intrinsic limitations, resulting from the difficulty of obtaining a mechanistic understanding of the underlying process based on a finite number of realizations, as well as the computational resources necessary to perform a large number of simulations in multi-component systems.

The benefits and limitations of stochastic and deterministic models cannot be determined *a priori*, as the appropriateness of each approach is constrained by the question under investigation and the assumptions of the model. For instance, from the perspective of an individual cell, fluctuations on PCN may be critical and therefore segregational noise would play an important role and therefore stochastic models would be more appropriate. In contrast, in a large bacterial population, noise in PCN is averaged and a deterministic model would be more convenient to describe the population dynamics of the population. Indeed, despite the intrinsic noise associated with replicating, segregating, and transferring plasmids, deterministic models based on ODEs have provided a conceptual framework that has been very successful describing the population dynamics observed in experimental model systems characterized by dense cultures and well-mixed environments, as well as producing testable predictions that have been used to design new experiments.

A limitation of most studies presented in this manuscript is that models focus on understanding plasmid dynamics occurring in simple experimental microcosms, like batch cultures or chemostats. But recent advances in genomic technologies and bioinformatic algorithms are beginning to shed light on the influence of plasmids and other MGEs in natural environments (Smalla et al., 2015; Li et al., 2020), progress that contrasts with the scarcity of theoretical models aimed at providing a mechanistic understanding of the role of plasmids in polyclonal populations. Therefore a fundamental problem in plasmid biology remains, and for which mathematical modeling could aid in finding a solution: what is the effect of MGEs in the structure and function of complex microbial communities? This is, of course, a very difficult question to address, mainly because it spans multiple levels of complexity (Paulsson, 2002). Indeed, W. Eberhard stated more than 30 years ago: “*As already noted by other authors* (Hardy, 1975; Broda, 1979; Dawkins, 2016), *analyses of plasmid evolution entail simultaneous consideration of selection acting at several different levels of reproduction, including genes, transposons, plasmids, chromosomes, cells, and clones*” (Eberhard, 1990). Decades later, many conceptual advances have been made in plasmid biology, including the realization of the importance of another level of selection: plasmids as ecological drivers of mixed bacterial populations and as promoters of community-level evolution (van Vliet and Doebeli, 2019; Doulcier et al., 2020).

Moreover, recent *in vivo* studies are beginning to track evolution in real-time inside complex communities (Ramiro et al., 2020) and to identify DNA transmission events directly from the microbiota (Munck et al., 2020). This is a very challenging task, although several quantitative methods used to precisely measure rates of HGT across multiple complexity scales have been proposed (Moralez et al., 2021). Then another challenge for the future is to produce data-driven models that incorporate high-throughput data obtained with high temporal and spatial resolution into predictive models of plasmid dynamics. We believe that the mathematical and computational tools necessary to include this information are yet to be developed, but most likely will not result from scaling-up systems of ODEs to consider thousands of equations with millions of parameters. The reason most studies discussed in this review have restricted to study simple model systems is not because of lack of interest or computational power, but because the analysis of simple models allows for fundamental insights that are difficult to obtain using more complex models.

Another problem associated with population dynamic models is the difficulty of estimating parameter values for individual strains embedded in mixed populations, in part because of empirical constraints (bacteria are not culturable, metabolic interactions are complex and distributions of fitness effects are heterogeneous), as well as due to theoretical limitations (interactions are highly non-linear and parameters can have identifiability issues). Despite these difficulties, or perhaps precisely because of these challenges, the future of mathematical modeling to study plasmid dynamics is a promising area of research, as illustrated by the large number of studies published in the past few years that have used mathematical modeling to study the ecological and evolutionary forces driving plasmid dynamics in bacterial populations.

Finally, as discussed previously, the introduction of mobile elements encoding for drug resistance genes into clinical environments can produce plasmid-related outbreaks of antimicrobial-resistant pathogens (Mayer, 1988). In this scenario, it could be argued that the responsibility for the outbreak lies not on a particular bacterial strain but on a plasmid that is shared between different hosts and, therefore, the drug resistant problem could be viewed from a plasmid-centric perspective (Wang and You, 2020; Baquero et al., 2021). We believe that mathematical modeling and computer simulations provide powerful tools to control the spread and evolution of plasmid-encoded drug resistance genes and, in the future, maybe even to propose new therapeutic avenues that control plasmid-driven antibiotic resistance.

## Author Contributions

All authors contributed to the literature review, analysis of the models presented, and writing of the manuscript.

## Conflict of Interest

The authors declare that the research was conducted in the absence of any commercial or financial relationships that could be construed as a potential conflict of interest.

## Publisher’s Note

All claims expressed in this article are solely those of the authors and do not necessarily represent those of their affiliated organizations, or those of the publisher, the editors and the reviewers. Any product that may be evaluated in this article, or claim that may be made by its manufacturer, is not guaranteed or endorsed by the publisher.

## Abbreviations

HGT: horizontal gene transfer
MGE: mobile genetic element
PCN: plasmid copy number
TA: toxin-antitoxin system
ODE: ordinary differential equation
PDE: partial differential equation
IBM: individual-based model
PSK: post-segregational killing.
