# Supplementary material for: Mathematical Models of Plasmid Population Dynamics
Source: Front Microbiol. 2021 Nov 4;12:606396. doi: 10.3389/fmicb.2021.606396 (PMC8600371; doi:10.3389/fmicb.2021.606396)
Supplement: Supplementary file 1 [file Table_1.pdf]

	Data	ODE	IBM	PDE	Other	Stochastic	Deterministic	Spatial	Topic
Novick and Hoppensteadt 1978		■			■	■	■		Plasmid replication
Lee and Bailey, 1984	x	■					■		Plasmid replication
Nordström et al. 1984		■					■		Plasmid replication
Seo and Bailey 1985		■		■		■			Plasmid replication
Bremer and Lin-Chao 1986	x	■					■		Plasmid replication
Greenhalf et al. 1989					■	■			Plasmid replication
Keasling and Palsson 1989	x	■					■		Plasmid replication
Müller et al. 1991					■	■			Plasmid replication
Brendel and Perelson 1993	x	■					■		Plasmid replication
Leipold et al. 1994	x	■					■		Plasmid replication
Kuo and Keasling 1996	x	■				■			Plasmid replication
Goss and Peccoud 1998		■				■			Plasmid replication
Paulsson and Ehrenberg 1998					■	■			Plasmid replication
Paulsson and Ehrenberg 2001		■				■	■		Plasmid replication
Watve et al. 2010		■					■		Plasmid replication
Field and Summers 2011					■	■			Plasmid replication
Ishii et al. 1978	x				■	■			Plasmid segregation
Nordström et al. 1980	x				■	■			Plasmid segregation
Nordstrom and Austin 1989					■		■		Plasmid segregation
Hsu and Chang 2019	x				■	■			Plasmid segregation
Münch et al. 2019	x	■					■		Plasmid segregation
Stewart and Levin 1977		■					■		Plasmid stability
Levin et al. 1979		■					■		Plasmid stability
Levin and Stewart 1980		■					■		Plasmid stability
Nordström and Aagaard-Hansen 1984	x				■		■		Plasmid stability
Ataai and Shulert 1987	x	■					■		Plasmid stability
Cooper et al. 1987	x	■					■		Plasmid stability
Lenski and Bouma 1987	x	■					■		Plasmid stability
Stephanopoulos and Lapudis 1988		■			■		■		Plasmid stability
Summers 1991					■		■		Plasmid stability
Mongold 1992		■					■		Plasmid stability
Macken et al. 1994		■					■		Plasmid stability
Bergstrom et al. 2000		■					■		Plasmid stability
Ganusov and Brilkov 2002	x	■					■		Plasmid stability
Hsu and Tzeng 2002		■					■		Plasmid stability
Dionisio 2005		■					■		Plasmid stability
Imran et al. 2005		■		■			■	■	Plasmid stability
Novozhilov et al. 2005		■			■	■	■		Plasmid stability
Mochizuki et al. 2006		■					■	■	Plasmid stability
Song et al. 2006		■		■			■		Plasmid stability
De Gelder et al. 2007	x	■				■			Plasmid stability
Lili et al. 2007		■					■		Plasmid stability
Ponciano et al. 2007	x	■				■	■		Plasmid stability
De Gelder et al. 2008	x	■				■			Plasmid stability
Svara and Rankin 2011		■					■		Plasmid stability
Yuan et al. 2011		■					■		Plasmid stability
Kentzoglanakis et al. 2013					■	■			Plasmid stability
Tazzyman and Bonhoeffer 2013					■	■			Plasmid stability
Yurtsev et al. 2013	x	■					■		Plasmid stability
San Millan et al. 2014	x	■					■		Plasmid stability
Harrison et al. 2015	x	■	■			■	■		Plasmid stability
Peña-Miller et al. 2015		■					■		Plasmid stability
Harrison et al. 2016			■			■			Plasmid stability
Werbowy et al. 2017	x				■	■			Plasmid stability
Hall et al. 2017			■			■			Plasmid stability
Lopatkin et al. 2017	x	■					■		Plasmid stability
Werisch et al. 2017			■			■			Plasmid stability
Grover and Wang 2019		■					■		Plasmid stability
Zwanzig et al. 2019		■					■		Plasmid stability
Gama et al. 2020		■					■		Plasmid stability
Jordt et al. 2020	x	■				■			Plasmid stability
Wang and You, 2020	x	■				■	■		Plasmid stability
Alonso-del Valle et al. 2021	x	■				■	■		Plasmid stability
Freter et al. 1983	x	■					■		Plasmid transmission
Lundquist and Levin 1986	x	■					■		Plasmid transmission
Clewlow et al. 1990	x	■					■		Plasmid transmission
Simonsen et al. 1990	x	■					■		Plasmid transmission
Simonsen 1991	x	■					■		Plasmid transmission
Macdonald et al. 1992	x	■					■		Plasmid transmission
Smets et al. 1993	x	■					■		Plasmid transmission
Beaudoin et al. 1998	x			■			■	■	Plasmid transmission
Licht et al. 1999	x	■					■		Plasmid transmission
Landis et al. 2000	x				■	■			Plasmid transmission
Smith 2001		■					■		Plasmid transmission
Lagido et al. 2003	x				■	■		■	Plasmid transmission
Gregory et al. 2006			■			■		■	Plasmid transmission
Krone et al. 2007	x		■			■		■	Plasmid transmission
Massoudieh et al. 2007	x			■			■	■	Plasmid transmission
Gregory et al. 2008			■			■		■	Plasmid transmission
Nogueira et al. 2009	x	■					■	■	Plasmid transmission
Philipsen et al. 2010	x	■				■			Plasmid transmission
Zhong et al. 2010	x	■					■		Plasmid transmission
Mc Ginty et al. 2011		■					■	■	Plasmid transmission
Merkey et al. 2011			■			■		■	Plasmid transmission
Rankin et al. 2011					■		■		Plasmid transmission
Volkova et al. 2012	x						■		Plasmid transmission
Levin et al. 2014	x	■					■		Plasmid transmission
Ahmad et al. 2015		■					■		Plasmid transmission
Baker et al. 2016		■					■		Plasmid transmission
Lopatkin et al. 2016	x	■				■	■		Plasmid transmission
Cazer et al. 2018		■					■		Plasmid transmission
Campos et al. 2019			■			■			Plasmid transmission
Klümper et al. 2019	x	■					■		Plasmid transmission
Tepekule et al. 2019		■				■	■		Plasmid transmission
Ledda et al. 2020	x				■	■			Plasmid transmission
Leon-Sampedro et al. 2020	x				■	■			Plasmid transmission
van Dijk et al. 2020		■	■			■	■	■	Plasmid transmission
Lopez et al. 2021	x	■					■		Plasmid transmission
Condit and Levin 1990	x	■					■		Plasmid evolution
De Gelder et al. 2004	x	■				■			Plasmid evolution
Joyce et al. 2005	x				■	■			Plasmid evolution
Mc Ginty and Rankin 2012		■			■		■		Plasmid evolution
Mc Ginty et al. 2013		■			■		■		Plasmid evolution
Ledda and Ferretti 2014				■			■		Plasmid evolution
Tazzyman and Bonhoeffer 2014					■	■			Plasmid evolution
Tazzyman and Bonhoeffer 2015		■					■		Plasmid evolution
Loftie-Eaton et al. 2016	x	■			■	■			Plasmid evolution
Rodriguez-Beltran et al. 2018	x	■					■		Plasmid evolution
Ilhan et al. 2019	x				■	■			Plasmid evolution
Mei et al. 2019	x				■	■			Plasmid evolution
Rodriguez-Beltran et al. 2019	x				■	■			Plasmid evolution
Santer and Uecker 2019		■			■	■	■		Plasmid evolution
Lehtinen et al. 2021		■					■		Plasmid evolution
